# Gih (Qi): Beyond Affordance

**DOI:** 10.3389/fpsyg.2017.00556

**Published:** 2017-04-11

**Authors:** Yang Lee, Robert E. Shaw, Zheng Jin

**Affiliations:** ^1^Center for the Ecological Study of Perception and Action, University of Connecticut, MansfieldCT, USA; ^2^Institute of Educational Sciences, Zhengzhou Normal UniversityZhengzhou, China

**Keywords:** Gih (Qi), affordance, mind–body problem, eastern philosophy, ecological psychology, perception and action, embodied cognition

## Abstract

Ancient Eastern thought posited the ontological integration of the “mind-body world”. The body-mind syncretism was a foundational precept in Eastern philosophy in which “Gih” (“Qi”) was considered the basic entity of the universe and the human being. This study attempts to build a meta-theory and to demonstrate empirical designs for Gih, discussing the problems of the mind and body, or the subject and object, compared with the concept of “affordance” proposed by ecological approaches. The notion of Gih extends beyond that of affordance in that Gih activates a psychosomatic process between the physical condition and the mental state that facilitates the bi-directional interactions between subject and object. Therefore, the concept of Gih integrates mind and body, providing a means of comparing Eastern and Western philosophical systems.

## Introduction

The concept of “affordance” proposed by James J. Gibson (1904–1979) pioneered the field of ecological psychology, defying the conceptual limitations of indirect realism reliant on constructive processes of representations or ideas in perceptual processes (Gibson, 1968, 1979). Ecological psychologists after Gibson (e.g., Turvey and Shaw, 1979, 1995; Shaw and Turvey, 1981; Turvey, 1992) further revised the concept of affordance (also see Turvey and Carello, 2012, for further discussions). They suggested that affordance underlies the coordination of perception and action. While raising some controversial issues, Lee et al. (2012) conducted experiments that demonstrated that perceptual scales can be varied to hit-ableness. For example, a shooter can perceive a target as enlarged when he or she hits it (a similar result is reported in Soccer by Jin and Lee, 2013). How can this result occur? This question is commonly subsumed under the philosophical issue of the “natural kind or proper observable” (Millikan, 1999; Ellis, 2001), for which perception serves as empirical evidence. Focusing on embodied cognition (e.g., Glenberg, 2010; Davis and Markman, 2012; Glenberg et al., 2013), studies integrating the perspective of subject–object can promote the concept of affordance. A comparable paradigm from Eastern philosophy explains the relationship between action adjustment and object estimation in a way that might be independent of the physical action that makes affordances available.

This study proposes to use the concept of “Gih (Qi)”^1^ in place of “affordance”. Gih, as conceptualized in Eastern philosophy, coordinates perception and action (see Lee et al., 2007; Jin et al., 2015; Lee and Shaw, unpublished, for further discussions). For example, Gih can be manifested in practicing a martial art, such as sword matching. In this case, an artist activates Gih, matching his or her opponent to react to attacks and defenses that are concretized where andwhen he or she catches the other opponent’s break. The artists are subject to a potential activation, such as Gih, which is attuned to the situation in which the perception-action occurs. This paper introduces and refines the concept of Gih to elaborate how perception is coordinated with action within this conceptual framework compared with that of affordance. The terms “Gih” and “affordance” were originally defined as follows:

*Gih is formed* (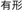) *through action for change* (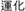) *to make heaven* ( 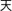 *refers to nature or object) and human* (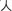 *refers to organism or subject) coordinated* (—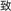). (Choi, 1857: 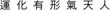 — 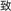).

*Affordance transcends the dichotomy of subjective-objective and helps us to understand its inadequacy. It is both physical and psychical yet neither physical nor psychical* (Gibson, 1979).

## Metaphysical Review

What is real resides in neither only the mind nor only the material (i.e., the body or matter), but in the relation between the two. The metaphysics of the mind and body have been controversial in philosophy since Rene Descartes (1596–1650) proposed the notion of “dualism”. Philosophical discussions on the limitations of the dichotomy (for a review, see Kim, 1996; Ravenscroft, 2005) have been revised to include “parallelism”, which was examined by Nicolas Malebranche (1638–1715), thereafter regressing into two types of “monism”, namely “materialism”, which was advocated by David Hume (1711–1776), and “idealism”, which was advanced by George Berkley (1685–1783). The metaphysics to integrate these paradigms constituted “the third paradigm”, proposed by Benedict de Spinoza (1632–1677) and Gottfried von Leibnitz (1646–1716) in the era of rationalism and subsequently renewed by existentialists such as Edmund Husserl (1858–1938) and Martin Heidegger (1889–1976) as well as analytic philosophers of language, guided by Peter Frederick Strawson (1919–2006). So-called third paradigms share the premise that the mind and body form two aspects of experience that originate from one entity, the third entity.

Given the above trajectory of philosophical thought, metaphysics engages with the problems of perception between a subject and an object. Theories of perception have been divided into empiricist and idealist. Empiricists assert that perceptual processes are guided by representations, which have been incorporated into experience. Idealists counter this assertion by arguing that perception is organized by a priori category in the mind that does not refer to experience. The third paradigm proposes to resolve the controversy between empiricists and idealists. For example, ecological approaches developed the concept of affordance, embracing certain concepts discussed in *quantum theory* to characterize the interaction between psychology and quantum mechanics either theoretically or empirically (Turvey, 2012; Shaw and Kinsella-Shaw, 2015).

Similarly, to Western philosophy, Eastern philosophy has discussed the problems between the person and the environment. Here, the person is the subject, and the environment is the object. The subject corresponds to the mind, and the object corresponds to the material. Thus, Eastern thought, which follows two main tracks, Taoism and Confucianism, has been concerned with the problem of how people can live as person–like in the environment (Pyung, 1934/1999; Feng, 2009; Tang, 2009; also see Yang, 1993, for further discussions). It is agreed that the environment exhibits natural laws and fates to which people accordingly try to adapt. Furthermore, Eastern philosophy is based on a third paradigm. Confucianism postulates two basic third entities, Gih and Lih (for a review, see Huh, 2004). Hwoang Lee (1501–1570) proposed that “Gih activated to implement Lih”. This statement implies that Gih is a force or a potential that motivates an event, and Lih is a reason or a purpose to be directed. Daeseung Gi (1527–1572) revised this understanding of the two entities, writing: “Gih is manipulated, and Lih is inferred”. Philosophers thus differentiate between the two basic entities of Gih and Lih.

Expressed in more scientific terminology, compared with the classic discussions, Hangi Choi (1803–1877) renewed a theory of Gih through the discipline of Gih (“Gihology”), which derives from Confucianism and Taoism but is also influenced by the Western disciplines, such as the electromagnetic theory of Newtonian physics (Choi, 1857). First, as the quotation at the end of the Introduction states, Gih is defined as “action for change (potential activated)”. Second, Gih is activated before it is “formed to be observed”. Third, Gih helps coordinate the human and the environment. According to this discussion and as elaborated by Lee et al. (2007) and Lee and Shaw (unpublished), the concept of Gih is comparable with but more advanced than the concept of affordance developed by ecological approaches (see Jin and Lee, 2013; Jin et al., 2015; Jin et al., 2016, for further suggestions).

## From Affordance To Gih

The concept of affordance is well known in ecological approaches as a concept for explaining the coordination of perception and action. A refinement of this concept or a substitution with a new concept is nonetheless more productive for facilitating further discussions in philosophy and biological processes. According to Gibson (1979), affordance discloses a relationship between a subject and an object, or the “psychical and physical”, respectively. This concept is a higher-level concept. To explain the concept at a more concrete level, the two directions must be understood as subject-to-object and object-to-subject. Later, ecological psychologists questioned whether this concept adequately explains a subject’s inclinations or an object’s properties. Whereas some scholars have criticized the implications of the concept, other scholars have defended it (for a review, see Michaels, 2003), contending that affordance connotes the object’s property, directed as object-to-subject, and the subject’s inclination, directed as subject-to-object. Therefore, the subject’s “effectivity” or some ability of action has been proposed (Michaels and Carello, 1981; Shaw et al., 1982). Other academics have proposed “intentionality” for action (Shaw, 2001) and “relation” (Chemero, 2003) to define an animal’s ability to interact with aspects of the environment. Finally, some definitions that were extended to include “meaningfulness” were proposed but criticized for being ambivalent in sensible testing (Michaels, 2003).

Thus, it remains controversial whether the revisions of affordance can free the concept from its original limitations. First, the means by which potential effectivity can be activated should be determined. Direction indicates what is implied with a bi-direction relation, such as subject-to-object or object-to-subject (e.g., Dotov et al., 2012). Second, how can potential effectivity be activated in a situation? Is effectivity primed by the mind, matter (or the body) or both? Third, it is understood that affordance is coextensive with information. Is it possible that affordance and information are not differentiated in concept and reality (c.f., Fajen et al., 2009)? In proposing a third paradigm, it is necessary to specify the entity that subordinates both the mind and the material and thus affects both the subject and the object. Therefore, this paper argues that one such third entity, Gih, as discussed by Choi (1857) and by Lee and Shaw (unpublished), is defined as potential activation. The argument is threefold. First, Gih is specified, not as static potential, but as activation without purpose. Second, Gih specifies bi-directional interactions between subject and object. Third, Gih is psychosomatic, thus relating the mind and the body. These three propositions indicate that when refined, the concept of Gih extends the understanding of the coordination of perception and action beyond that proposed by the concept of affordance.

Gih is demonstrated in the genealogy of the words used in daily living as “blood-Gih” and “mind-Gih” (Lee and Shaw, unpublished). Blood-Gih denotes passion and intention and represents the activation from body (blood) to mind (emotion). By contrast, mind-Gih corresponds to the health and physical conditions as instances of activation from mind to body. Therefore, blood-Gih and mind-Gih possess features that can be characterized as activation and together represent a bi-direction of Gih, mind-to-body and body-to-mind. The question arises regarding how the blood or mind is compounded with Gih to direct the other. Both the blood and the mind are hypothesized to attain their relative properties. It is through psychosomatics that the body (blood) works with the mind. As the term psychosomatic implies, Gih attempts to incorporate both the mental and biological processes in terms of Eastern philosophy and Oriental medical science (Leslie and Young, 1992; Tateno, 1993). Thus, Gih performs work that is psychosomatic (see Lee and Shaw, unpublished, for further discussion).

There is a critical reason to separate Gih from affordance. As noted, Gih should be differentiated from Lih, which in contrast implies a kind of reason that serves what is intended (see Hwoang Lee and Daeseung Gi’s discussion in the above chapter “Metaphysical Review”). As established by Gibson and further developed by later ecological psychologists, affordance is embedded with information, which thus implies directing the effort of coordinating perception and action for a purpose. By contrast, Gih is an activation potential and thus is not directed, as it does not have purpose. Some philosophical perspectives suggest concepts similar to Gih. For instance, “Elan vital” was proposed as a “living force of no teleology” by Henri Louis Bergson (1859–1941) (Papanicolaou and Gunter, 1987).

Consider a hypothetical example to illustrate the differences between the understandings of perception and action that result from applying the concepts of Gih and affordance. Suppose a dog approaches a girl to bite her. The girl runs away from the dog. Explained with the concepts of affordance and information, the girl is informed that the dog is about to bite her, and she is afforded with running away from the situation. However, modeling the situation in terms of Gih, if the girl’s Gih is not activated, then she cannot run away and thus cannot prevent herself from being bitten by the dog. Hence, Gih activation is a prerequisite to what follows with purpose, such as running away. Gih is effectively neutral in purpose, though it can be activated. Consider a more dramatic situation, such as a case in which the girl’s Gih is highly activated. Suppose she encounters a high wall while being chased by the dog. She will fight the dog. At this moment, Gih is activated and is directed in terms of subject-to-object for the girl and object-to-subject for the dog and the wall, which also coordinates the mutual directions. Thus, the girl’s Gih activation is situational because the wall blocks her retreat. Gih is activated by and construed as a psychosomatic process such as a fear of biting or the valor of combat, respectively manifested as running or fighting. What is psychosomatic has the potential to activate and influence both the mind and the body, and its function is mediated by the hormonal metabolic and automatic nervous systems.

## Gih As Psychosomatic Open To Eastern Disciplines

Sword matching, the example mentioned in the Introduction, offers a better example than the previous example of the girl and the dog for comprehending Gih as a psychosomatic process. An artist (A) performs a more advanced technique, such as a counter-attack, on his or her opponent (B)’s body part (for example, B’s wrist), just after (B) starts to attack (A) (for example, A’s head). When (B) attacks (A)’s head, (B) must open his wrist, which is performed at a sufficiently slow pace for (A) to counter-attack, despite the wrist being a small part moving quickly. How can this counter-attack be explained? Lee et al. (2012) suggested that (A) could enlarge the small space of (B)’s hidden body part, the wrist, and lengthen the time of (B)’s wrist movement to succeed in a counter-attack in a second. Therefore, the perceptual scale of (A) was enhanced (see Jin and Lee, 2013, for generalized empirical evidence). Referring to a cognitive explanation, (A) is more skilled because of repeated practice. However, this model can explain only A’s skills, which are not specified in time and space but are evaluated based on average conditions. In terms of the Gih model, (A) has Gih as the psychosomatic potential that is attuned to the present situation of perception-action, which is activated by spiritual intention and coordinated by physical autonomic processes. Gih can be trained through martial arts practice and conceptualized as a type of physical and spiritual learning. However, in the above case of the weak girl who must fight against the cruel dog when blocked by the high wall, the level of Gih activation increases without training. Thus, Gih can be situationally activated for survival to release psychosomatic processes between the mind and body, thereby accommodating the person to the environment.

If Gih is understood in terms of the psychosomatic process, it makes sense that meditation practices and treatments performed in Oriental medical science are examples of the principle of Gih. Meditation practice relies on certain correct postures and a focused mind. For the technique to integrate the mind and body, it is advised that respiration should be controlled (e.g., Schure et al., 2008; Jung et al., 2010). Breathing is normally processed involuntarily but can be controlled voluntarily with training. The control of respiration activates the mediation between physical conditions—such as blood circulation, hormonal metabolism, and nerve activation—and mental states—such as consciousness, cognition, and emotion. Thus, respiratory control is a psychosomatic process. Oriental medical science can also be theorized as a means of circulating Gih. Practitioners diagnose patients by touching the blood waves of the body (ordinary, wrists), checking the strength and rate of respiration, observing the colors of the face, and noting other vital signs. The techniques used to treat the psychosomatic variable of Gih include techniques such as the treatment of natural medicines, hot steam stimuli (moxibustion), and needle stimuli (acupuncture). Positive results have been reported in scientific studies, despite some controversies (see Unschuld, 1985; Leslie and Young, 1992; Guan and Fan, 2002; Noble, 2009, for further discussions).

What can the consideration of Gih as a psychosomatic variable contribute to empirical analysis? With respect to experimental procedures, Gih must be measured through its effects on behaviors associated with the mind. The observable phenomena can then be attributed to certain scales in perception and action, which are described as the terminal measures. For the measures of perception and action, studies have analyzed the influences of only some physical variables. In experimental analyses, Lee et al. (2012) manipulated the conditions of an archer’s arm control. The treatment in the study conducted by Jin and Lee (2013) varied according to soccer players’ running speeds. In the previous example of the artist sword matching, treatment can similarly be varied based on patterns of footwork. Physical manipulation has been understood to be empirical. Despite the convenience of manipulation; however, such physical variables are insufficient because they do not account for mental events. Therefore, the intermediated variable must be manipulated at the psychosomatic level, which links physical states and mental processes.

For a psychosomatic design, respiration control can be proposed in the sword matching example. Martial artists are adept at catching attack points while the space or time to attack is enlarged or prolonged. These phenomena occur by way of the psychosomatic process of Gih enhancement, which is activated by respiration control. In terms of paradigms, Gih as a psychosomatic model can be construed as a “hologram” model, such as that proposed by Pribram (1991), who argues that classic paradigms have overly relied on analytic dispositions. Because Gih, which is manipulated by respiration and other available processes, is a psychosomatic variable, it must be reevaluated as holographic rather than analytic. Thus, the discussion of Gih can be extended to arrange the empirical variables in a hierarchy ranging from the physical variables through the psychosomatic variables to the mental variables.

Because the psychosomatic variable of Gih is institutionalized, what is disposed of in popular discussions of psychological questions should be elaborated and further clarified. Problems concerning communication in language, aesthetic evaluation, and social relations represent some instances of mediation between mind and matter, or subject and object. If what has been discussed in this paper regarding blood-Gih and mind-Gih is extended, the terms of speaker-Gih and listener-Gih, artist-Gih and appreciator-Gih, and social-Gih between the subject and object could be tentatively proposed, provoking further discussion.

## Conclusion

The concept of Gih is compatible with Gibson’s concept of affordance in some respects. In other respects, though, the two concepts differ. Gih is a potential activation that possesses not only physical properties but also a mental disposition, influencing both subject and object, and thus should be considered a third entity.

Along with the subsets “mind-Gih” and “body-Gih” (also called “blood-Gih”), the concept of Gih accounts for the bi-directional interactions between mind and body. Thus, the concept advances the understanding of the coordination of perception and action beyond that enabled by the concept of affordance as initially proposed (Dotov et al., 2012) and conforms to revised notions of intentionality and effectivity (Michaels and Carello, 1981). The role of information in enacting affordances implies that the latter is teleological. By contrast, Gih refers to the potential to activate mental and physical states and thus lacks purpose. Gih activation between the mind and body can be refined with training through meditation or respiration, which are known to control psychosomatic processes, thus influencing an involuntary mechanism through voluntary control. The possibility of such refinement remains a topic for discussion in Eastern philosophy and Oriental medical science.

Gih can be considered a psychosomatic variable that is located at the mid-level in a hierarchy of variables ranging from physical to mental and thus should be distinguished from affordance. Hence, Gih also passes the philosophical test of Occam’s razor, which demands no redundancy when scientific terms are created. Therefore, the refined term of Gih is not synonymous with affordance. Nevertheless, further discussion is required to discern whether the Gih concept could incorporate elements of physical psychology (Turvey and Carello, 2012), which would mark a theoretical advancement from the ecological approaches. Looking forward, the Gih concept accommodates communication processes, aesthetic feelings, and social relationships and may offer a way to integrate Eastern and Western traditions of thought concerning the coordination of perception and action.

## Author Contributions

YL: substantial contribution to the conception of the work; drafted the work. RS: substantial contribution to the conception of the work; substantial revision of the work. ZJ: substantial contribution to the conception of the work; substantial revision of the work. All authors contributed equally to this manuscript.

## Conflict of Interest Statement

The authors declare that the research was conducted in the absence of any commercial or financial relationships that could be construed as a potential conflict of interest.

## Footnotes

1 The term is spelled “” and pronounced “Gih” in Korean. It is written “” and is pronounced “Qi” in Chinese. In this article, the term *Gih* corresponds to *Qi*.
